# Anterior insula coordinates hierarchical processing of tactile mismatch responses

**DOI:** 10.1016/j.neuroimage.2015.11.030

**Published:** 2016-02-15

**Authors:** Micah Allen, Francesca Fardo, Martin J. Dietz, Hauke Hillebrandt, Karl J. Friston, Geraint Rees, Andreas Roepstorff

**Affiliations:** a Institute of Cognitive Neuroscience, University College London, London WC1N 3AR, United Kingdom; b Wellcome Trust Centre for Neuroimaging, University College London, London WC1N 3BG, United Kingdom; c Center of Functionally Integrative Neuroscience, Aarhus University Hospital, Aarhus 8000, Denmark; d Harvard University, Cambridge, MA, 02138, United States; e Interacting Minds Centre, Aarhus University, DK-8000 Aarhus C, Denmark

## Abstract

The body underlies our sense of self, emotion, and agency. Signals arising from the skin convey warmth, social touch, and the physical characteristics of external stimuli. Surprising or unexpected tactile sensations can herald events of motivational salience, including imminent threats (e.g., an insect bite) and hedonic rewards (e.g., a caressing touch). Awareness of such events is thought to depend upon the hierarchical integration of body-related mismatch responses by the anterior insula. To investigate this possibility, we measured brain activity using functional magnetic resonance imaging, while healthy participants performed a roving tactile oddball task. Mass-univariate analysis demonstrated robust activations in limbic, somatosensory, and prefrontal cortical areas previously implicated in tactile deviancy, body awareness, and cognitive control. Dynamic Causal Modelling revealed that unexpected stimuli increased the strength of forward connections along a caudal to rostral hierarchy—projecting from thalamic and somatosensory regions towards insula, cingulate and prefrontal cortices. Within this ascending flow of sensory information, the AIC was the only region to show increased backwards connectivity to the somatosensory cortex, augmenting a reciprocal exchange of neuronal signals. Further, participants who rated stimulus changes as easier to detect showed stronger modulation of descending PFC to AIC connections by deviance. These results suggest that the AIC coordinates hierarchical processing of tactile prediction error. They are interpreted in support of an embodied predictive coding model where AIC mediated body awareness is involved in anchoring a global neuronal workspace.

## Highlights

• A body-related network of thalamic, somatosensory, insular, cingulate, and prefrontal areas responded to tactile oddballs.
• Dynamic Causal Modelling revealed oddball stimuli modulated an ascending rostral-to-caudal hierarchy between these areas.
• Within this feed-forward flow, only anterior insula showed reciprocal increases in connectivity with somatosensory cortex.
• Subjective deviance detection ratings predicted the strength of feedback from prefrontal cortex to anterior insula
• These results provide preliminary evidence for the newly emerging theory of embodied predictive coding.

## Introduction

The body is our first point of contact with the world, forming the backdrop of all perceptual and emotional experiences. As the body's largest organ, the skin delineates an agent's boundaries, controls thermoregulation, and encodes information about the shape, intensity, and texture of bodily impressions. In general surprising tactile changes herald events of high motivational importance, including imminent threats (e.g., an insect bite) and pleasing social rewards (e.g., a caressing touch). Likewise, expectations and prior experiences colour our subjective perception, enhancing task-relevant features and suppressing others (Clark, 2013). Here we used Dynamic Causal Modelling of fMRI signals associated with unpredictable changes in the intensity of tactile stimuli. The ensuing changes in effective connectivity show how feed-forward and feed-back processes are integrated by the insula to support body awareness.

One framework for understanding how prior beliefs and tactile changes are integrated in the brain is found in recent theoretical proposals describing a union of embodied perception and predictive coding (Seth, 2013). Here a general theoretical consensus suggests that the integration of bodily changes with prior beliefs underlies the generation of affective awareness (Apps and Tsakiris, 2014, Barrett and Simmons, 2015, Seth et al., 2012). In particular, the anterior insula is thought to be crucial for the hierarchical processing of bodily information, integrating afferent thalamic and sensory inputs with top-down control signals arising in the prefrontal and cingulate cortex (Seth, 2013, Seth et al., 2012). This theory is supported by the unique structural and functional characteristics of the insular cortex as a core neuronal hub that causally regulates the interaction of sensory, attentional, and default mode networks (van den Heuvel et al., 2012, Menon and Uddin, 2010, Sridharan et al., 2008). This high centrality equips the region with the unique ability to integrate diverse cortical and subcortical inputs, as supported by both a variety of multi-sensory and thalamic posterior inputs and anterior projections to cingulate, prefrontal, and brain-stem nuclei (Craig, 2003, Craig, 2005, Ullsperger et al., 2010). The right AIC in particular is richly interconnected with primary visceral and somatosensory areas such as posterior insula and somatosensory cortex (Cerliani et al., 2012, Chang et al., 2013), anticipates the sensory and affective consequences of touch (Lovero et al., 2009), and has been described as a central node in a right-lateralized body-related network (Craig, 2005).

This unique anatomical and functional profile suggests that the right anterior insula supports bodily and affective awareness by integrating ascending information from thalamic and sensory sources with descending predictions from the prefrontal and cingulate cortex (Seth, 2013, Seth et al., 2012). Neurobiological theories inspired by predictive coding generally ascribe functional asymmetry to ascending and descending connections (Bastos et al., 2012). Evidence for this distinction is found for example in a recent study demonstrating that whereas ascending prediction errors are communicated by increases in fast gamma oscillations, descending predictions are instead carried by slower theta and alpha oscillations (Bastos et al., 2015). In general under active inference, perceptual awareness is argued to depend upon the inversion of a generative model that encodes error-minimizing expectations, which then constrain activity in lower-order regions via top-down cortical feedback (Friston, 2010, Friston et al., 2012, Rao and Ballard, 1999).

With respect to oddball tasks, mismatch responses elicited by the comparison of standard stimuli to unexpected deviants are computationally well fit by prediction error minimization schemes (Garrido et al., 2007, Garrido et al., 2008, Lieder et al., 2013a, Lieder et al., 2013b), and are mediated by asymmetrical changes in intrinsic and extrinsic effective connectivity within a hierarchical network (Dietz et al., 2014, Garrido et al., 2008, Garrido et al., 2009). Recently, tactile awareness been shown to rely on the existence of reciprocal effective connectivity within the somatosensory hierarchy (Auksztulewicz et al., 2012). Tactile mismatch specifically depends on hierarchical Bayesian inference in somatosensory and limbic areas (Ostwald et al., 2012): EEG responses elicited in a roving tactile oddball paradigm were modelled to demonstrate that somatosensory and cingulate mismatch responses encode Bayesian Surprise, a measure of the amount of prediction error (Baldi and Itti, 2010, Ostwald et al., 2012). It is currently unknown how tactile deviants regulate effective connectivity within the right-lateralized body awareness network (Craig, 2005). This study therefore aimed to investigate how attending to bodily changes modulates anterior insula effective connectivity within the tactile hierarchy.

To do so, we modelled BOLD mismatch responses in a roving oddball paradigm using Dynamic Casual Modelling for fMRI. In particular, we derived three connectivity hypotheses from the above. First, if tactile information is processed hierarchically, one should expect a general pattern of increased forward connections in response to unpredicted tactile stimuli, reflecting their role in conveying prediction error. Second, and most crucially for the present experiment, we expected increases in both forwards and backwards insular connections; such a result would demonstrate that the AIC regulates the activity of both cingulo-prefrontal and somatosensory responses (Seth, 2013, Seth et al., 2012). Finally, if top-down modulation of anterior insula responses underlies our ability to attend to and perceive bodily changes, we expected subjective ratings about stimulus changes to correlate with modulation of descending connections to the anterior insula during oddball responses. Here, we provide evidence for each of these hypotheses using the roving tactile oddball paradigm and Dynamic Causal Modelling for fMRI.

## Methods

### Participants

Thirty-eight healthy participants (16 males) were recruited from Aarhus University and the surrounding community. Inclusion criteria required that all participants were between the ages of 21 and 45 years, right handed, free from medications that could affect the BOLD signal (psychiatric, blood pressure or heart medication, etc.), physically and mentally healthy, and meeting standard MRI safety inclusion criteria (lack of claustrophobia, metallic implants, etc.). All participants gave verbal consent and visited the MRI laboratory at Aarhus University Hospital for approximately 2 h in total, and received a 300 DKK (approx. €40) reimbursement for their participation. All experimental procedures were conducted with approval from the local ethics committee (De Videnskabsetiske Komitéer for Region Midtjylland) in accordance with the Declaration of Helsinki. 8 participants in total were excluded from preliminary data analysis—one for excessive motion during scanning, 6 for poorer than chance behavioural performance (see Roving Somatosensory Oddball Task for further details), and one for failure to acquire pulse regressors. The final sample for the fMRI analysis included 30 participants (14 males) with a mean age of 24.5 years (SD = 3.2).

### Roving Somatosensory Oddball Task

To manipulate tactile probability while controlling for stimulus intensity and attention, we utilized a Roving Somatosensory Oddball Task (RSOT) in which trains of stimuli randomly switch between high and low intensity after a variable number of repetitions (Garrido et al., 2008, Ostwald et al., 2012). In the present study, stimuli were delivered in trains of varying from 3 to 7 repetitions. Stimuli consisted of single electrical pulses of 50 μs duration and 2000 ms interstimulus interval. Following each repetitive train, the stimuli switched between low or high intensity, where low intensity trials corresponded to a single pulse at twice the perceptual threshold, and high intensity trials consisted of two pulses identical to the single delivered in rapid succession (100 ms inter-stimulus interval).

**Fig. 1:**
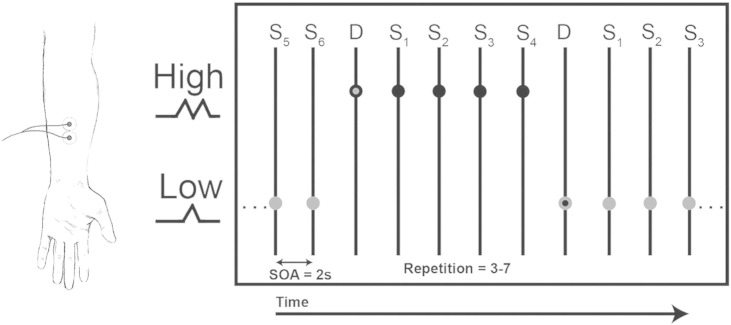
Schematic depicting experimental setup and example stimulus train. Participants received mild somatosensory electrical stimulation (50 μs pulse) at twice sensory threshold on the median nerve of the left forearm. Subjective intensity was manipulated by switching between single pulse (bottom-row) and double pulse (top-row) trials. Double pulses were identical to single pulses, with a 100 ms interstimulus interval. Repetitions varied randomly from 3 to 7 standard stimuli before switching to the alternate stimulus type, with repetition counts randomly sampled from uniform multinomial distribution. The first stimulus of each train corresponded to a deviant (D), whereas the following repetitions were defined as standards (S1, S2, …, S6). For our fMRI analysis, we modelled the deviant trials and the third repetition as standard (see Methods for more details).

This stimulation protocol resulted in a sensation of a mild tickle or vibration that was not reported as painful by any participant. The first stimulus of each new train was modelled as the “Deviant” (D), and the third repetition in a train as the “standard” (S). Standards were repeated between 3 and 7 times by sampling at random from a uniform multinomial distribution with outcomes {3, 4, 5, 6, 7}, generating an unpredictable stimulus sequence. Participants received a total of 158 deviant and 640 repeated stimuli (of which 158 stimuli were selected as standards). All stimuli were delivered to the median nerve of the left forearm using two MR-safe ECG electrodes placed approximately 2.5 cm apart and a constant current stimulator (DeMeTec, Langgoens, Germany). See Fig. 1 for an overview of the experimental set-up and sample stimulus train.

After placement in the scanner, participants' individual perceptual thresholds were determined using an adaptive staircase procedure prior to scanning. The staircase consisted of a one-up/three-down procedure, where step size was reduced every two reversals until reaching a total of 8 reversals. The sensory threshold was thus calculated by averaging the stimulus intensities at the 8 reversals. Stimuli for the subsequent oddball task were then delivered at twice this sensory threshold, eliciting a mild tactile sensation. After thresholding, participants completed a short practice version of the oddball task, and continued to the main experiment after indicating that the task instructions were fully understood. All participants completed approximately 28 min of the RSOT during fMRI acquisition.

Pilot studies with this stimulation protocol revealed that – as intended – the double stimulation was perceived as slightly more intense than single trials. To control attention, participants were instructed to silently count all stimuli switches throughout the entire task duration, in a standard ‘active’ counting task (Garrido et al., 2009). This manipulation encourages participants to exert equivalent attentional effort to both deviant and standard trials (as the occurrence of deviants is unpredictable). Participant switch counts were then recorded at the end of the imaging session to ensure compliance. Six participants reporting switch counts 60% above or below the true total (i.e., poorer than chance performance) were excluded from further analysis. Overall switch count accuracy of the remaining participants was extremely high (mean accuracy 99%), suggesting successful attentional control and task participation.

Following the scan, participants completed a debriefing inquiring about the nature of the perceived stimuli (e.g., painful or non-painful). Participants also rated “the felt intensity” of each stimulus type (i.e., low and high), and “the difficulty detecting stimulus changes from low to high” or “from high to low”, on visual analogue scales with 0 marked as ‘not at all intense/difficult’ and 100 labelled as ‘very difficult/intense’. The adaptive staircase procedure, the RSOT and the post-scan ratings were implemented in Psychopy (v1.76.00) (Peirce, 2007).

### Data acquisition and pre-processing

fMRI data were collected in a single continuous session of approximately 28 min, totalling 1109 volumes. All brain measurements were acquired on a Siemens Trio 3 T scanner, using a 32-channel head coil. For fMRI, 31 slices were acquired in ascending order using a gradient echo planar sequence with echo time 30 ms, voxel size 3 × 3 × 3 mm in a 64 × 64 mm field of view, repetition time = 1.54 s. Slices were manually positioned to ensure full coverage of somatosensory cortex, anterior insula, prefrontal cortex, and thalamus, flip angle = 90°. A T1-weighted MPRAGE structural image (0.98 × 0.98 × 1.00 mm resolution, FoV = 256 × 256 × 176, TR = 1.9 s) was collected after the EPI sequence. B_0_ field maps (2.19 × 2.19 × 2.50 mm resolution, 96 × 96 × 60 FoV, TR = 1.43 s) were collected using a gradient echo sequence. To control for physiological BOLD-signal confounds, cardiac cycles were recorded in synchrony with EPI acquisition using an infrared pulse oximeter on the participant's right index finger.

### fMRI analysis

MRI data were analysed using Statistical Parametric Mapping (SPM8 for GLM analysis and SPM12b for DCM, http://www.fil.ion.ucl.ac.uk/spm). Each participant's 1109 EPI images were corrected for geometric distortions caused by susceptibility-induced field inhomogeneity. This was done using a combined correction for both static distortions and changes in those distortions caused by head motion (Andersson et al., 2001, Hutton et al., 2002). Static distortions were calculated using the FieldMap toolbox to process each participant's B_0_ field map (Hutton et al., 2004). EPI images were then realigned, unwarped, and co-registered to the participant's anatomical scan. The anatomical images were processed using the unified segmentation procedure implementing tissue segmentation, bias correction, and spatial normalization (Ashburner and Friston, 2005); derived normalization parameters were then applied to the EPI images. Finally, the images were smoothed using a 6 mm full-width at half-maximum Gaussian kernel, and resampled to 3 × 3 × 3 mm voxels.

To control for head motion and physiological BOLD signal confounds, serial correlations were modelled using a nuisance variable regression instead of the AR(1) SPM default, which has been shown to outperform the auto-regressive technique at faster TRs (Lund et al., 2006). In addition to the SPM8 standard discrete cosine set high pass filter (128 s cut off), this approach included 10 RETROICOR-derived regressors based on cardiac oscillations (Glover et al., 2000). We also included the full 12 parameter Volterra expansion of motion and motion history parameters to capture rigid body head movement related to subject motion and respiration (Friston et al., 1996).

To identify BOLD responses to somatosensory deviance, we applied a standard summary statistic approach (Worsley et al., 2002). This involved first identifying subject-level responses in a fixed-effects general linear model (GLM) for each participant's EPI time-series, and then passing the resultant contrast images to one-sample t-tests at the group (random-effects) level. To do so, at the first level we modelled Deviants (the first trial of a new stimulus intensity) and Standards (the third repetition following each Deviant) as separate event-related regressors convolved with the canonical hemodynamic response function. The remaining repetition trials (S2–S7) were not modelled, and thus served as an implicit baseline. The resulting contrast images were then passed to a random-effects one sample *t*-test over all participants, testing for a positive mean response. The resulting SPM was peak-corrected for multiple comparisons at a family-wise error rate *P_FWE_* < 0.05 using Gaussian random field theory (Worsley et al., 1996).

### Dynamic causal modelling

To address our connectivity hypotheses, we investigated the overall impact of surprising tactile changes on effective connectivity within the right lateralized network identified by our mass-univariate analysis. To do so, we used a Bayesian model reduction approach to model selection that searches large model spaces in an unbiased way (Friston and Penny, 2011, Rosa et al., 2012). This allowed us to search within a “full” or “parent” model – which contained all free parameters – for the best of all reduced models, with one or more parameters removed. Using the parameter estimates from the best model, we applied classical statistical tests to summarize changes in the strength of specific connections at the group level. We were thus able to make quantitative inferences about the strength and directionality of connections within an inclusive model space, while circumventing limitations regarding the combinatorial explosion of models (Lohmann et al., 2012).

In order to model the impact of oddball stimuli (D > S trials) on connectivity, we remodelled our first level (within-subject) design matrix into a single “all conditions” regressor encoding all trials (both deviants and 3rd standards) as “1”s. This condition was then parametrically modulated as a “1” for every deviant and a “− 1” for every standard trial. Note that this reformulation is simply a matter of convenience (i.e., to allow a contrast rather than a particular trial type to act as an input to DCM) and is statistically equivalent to our original within-subject model.

We then extracted BOLD timeseries from volumes of interest (VOI), based on the peak activations induced by oddballs (D > S at the group level). To this end we defined VOIs in the dorsal–posterior thalamus (TH) [MNI_xyz_ = 12, − 16, 10], somatosensory area 2 [MNI_xyz_ = 48, − 34, 49] (S1), anterior insula cortex (AIC) [MNI_xyz_ = 36, 20, 1], anterior mid-cingulate cortex (MCC) [MNI_xyz_ = 3, 23, 43], and middle frontal gyrus (MFG) [MNI_xyz_ = 36, 50, 22]. All anatomical labels at the extraction coordinates were confirmed using the SPM Probabilistic Anatomy Toolbox (Eickhoff et al., 2005).

VOI timeseries were then summarized in terms of the principal eigenvariate within a 6 mm spherical VOI centred on each participant's local maxima within 12 mm of the group maximum. Peak coordinates were plotted on a standard brain and inspected to ensure that all extracted timeseries were from the appropriate anatomical region of interest. For extraction, participant-level SPMs were thresholded at *p* < 0.05 uncorrected, voxel extent threshold *k* > 5 contiguous voxels. All timeseries were adjusted for confounding effects (i.e., nuisance covariates in the GLM). Given our interest in the right-lateralized body network and left-sided median nerve stimulation (Craig, 2005), all VOIs were taken from the right hemisphere. In five participants, regional VOIs from one or more regions could not be obtained, leaving 25 total participants for DCM analyses.

We chose a somewhat conservative approach to modelling the oddball effect by using the difference between deviants and standards as both a driving and modulatory input. This choice was based on the observation that, from the point of view of fMRI, the repeated presentation of stimuli every 2 s is effectively a steady state stimulus; in which haemodynamic responses are evoked by occasional deviants (relative to an arbitrary standard). This approach is conservative because condition-specific effects have two opportunities to express themselves (either as driving effects or by changing connectivity). This means that when we test for a modulatory effect (by removing it from the model), the driving effect could compensate for its absence (and vice versa). Heuristically, this is like including the driving effect as confound when testing for modulatory effects in general linear models (and vice versa), and ensures that any observed experimental modulations are not explained solely by driving input. The full model thus included the impact of deviants as a driving input to the thalamus, and additional modulatory deviance effects on all extrinsic (between node) and intrinsic (self) connections. The full model with extrinsic (fixed) connections between all nodes and deviant vs. standard modulations of all connections was then estimated in each participant using the variational Bayesian scheme implemented in SPM12. The neuronal dynamics were modelled with the following differential equation:

$$\frac{\delta x}{\delta t}=\mathrm{Ax}+\left(\sum_{k=1}^{I}u_{k}B^{k}\right)x+Cu\text{.}$$

The full model thus allowed changes in all A (condition independent) and B (modulatory or condition-dependent) parameters (with C mediating subcortical input at the thalamus). Bayesian model reduction (a.k.a. post hoc optimisation or network discovery) was used to compare all combinations of average (A) and condition-specific (B) connections (Friston and Penny, 2011, Rosa et al., 2012). Effectively, this removes (combinations of) redundant parameters or connections until model evidence stops increasing. Connections are ‘removed’ by setting their prior expectation to zero (with a prior uncertainty or variance of zero). This creates reduced models from the full or parent model. This furnishes posterior model probabilities (i.e., the probability that a model is the best explanation for the data) for all reduced models (including a null-model with no connections). Bayesian model reduction operates at the group level by pooling the evidence for each model over subjects (under fixed effect model assumptions). This simply entails summing the log evidence over subjects. The best model is then whichever has the highest evidence as determined by the Bayes factor; i.e., the ratio of evidence for the best vs. second best model (Penny et al., 2004). The parameter estimates under the winning model were then taken for each subject as summary statistics for classical random effect analysis at the group level. This involved performing one sample t-tests (with FDR correction for multiple comparisons).

Here, we focused on two connectivity hypotheses. First, we addressed our principal question about the pattern of insula connectivity by characterizing the deviant-dependent connectivity modulations. This was accomplished using one-sample t-tests over the 25 modulatory (B-matrix) parameters, *P_FDR_* < 0.05. Second, we separately assessed the relationship between individual differences in participants' perceived difficulty detecting sensory changes (i.e., the averaged post-scan difficulty ratings) and deviant-dependent modulation of each extrinsic connection (i.e., the 20 between-region B-matrix parameters). To do so, we conducted robust regression analyses using Tukey's Biweight with connectivity estimates predicting mean difficulty rating. This method was chosen over a least squares approach to protect against outlier values, which are a frequent issue in neuroimaging individual differences analyses (Poldrack, 2012). Regression *p*-values for each analysis were adjusted for multiple comparisons to a *P_FDR_* < 0.05. All ANOVA and one-sample *t-*test analyses were conducted in SPSS version 20 (IBM), and all FDR thresholds and robust regression analysis were calculated using MATLAB R2012b (Mathworks, Inc) and the FDR toolbox.

## Results

### Sample characteristics and nuisance regression

Excluding the 6 participants removed for poorer than chance performance, the average number of counted deviants was 156 (SD = 17) out of 158 total, corresponding to an average count accuracy of 99%. This result indicates that the majority of participants were able to comply with the task instructions, precluding major differences in attentional effort between standard and deviant trials. In the post scan debriefing, all participants reported that the stimuli were perceived as a non-painful mild touch or ‘tickle’ sensation. The average sensory threshold across participants was 12.22 mA (SD = 2.86). As a face-validation of our stimuli, we compared participant's intensity and difficulty ratings for low vs. high stimuli via paired-sample t-tests. Double stimuli (mean intensity rating = 57, SD = 21) were rated as significantly more intense than single stimuli (mean intensity rating = 46, SD = 16, mean difference = 11, SD = 19, *t_29_* = 3.4, p = 0.002). As no significant difference was found for the self-rated difficulty of discriminating single-to-double (mean difficulty rating = 34, SD = 26) or double-to-single trials (mean difficulty rating = 36, SD = 23, mean difference = − 1.5, SD = 17.5, *t*_29_ = − 0.5, *p* = .64), we averaged the two ratings from each participant to derive an index of the perceived difficulty detecting sensory changes. This measure was then used as an explanatory variable in our regression analyses with DCM modulatory parameters.

### Mass-univariate results

**Fig. 2:**
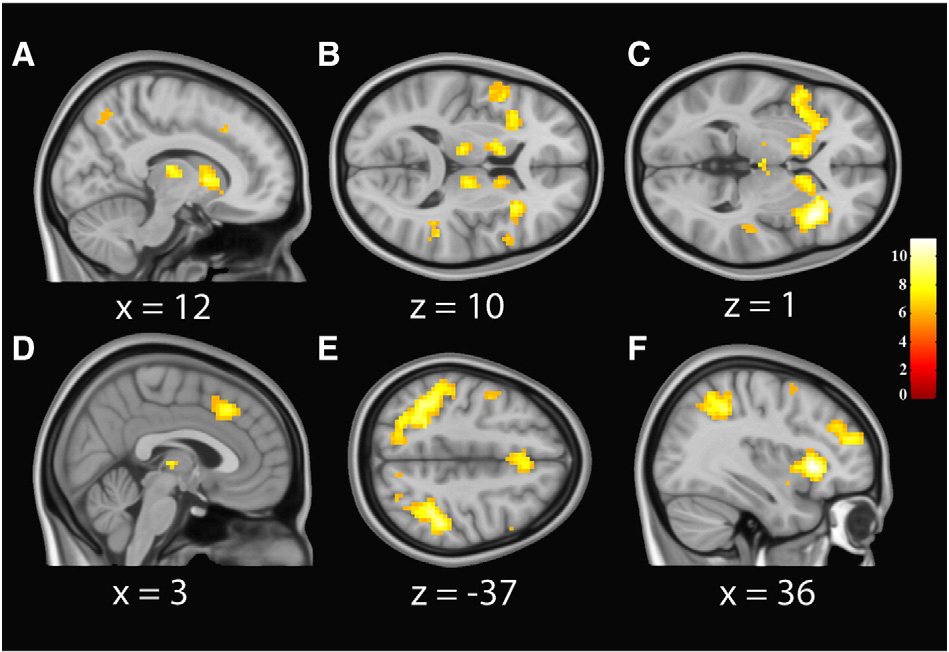
Significant BOLD activations for the deviant > standard contrast. From left to right, images are centred on the peak voxel extracted for each region modelled in the DCM; dorso-posterior thalamus (panels A and B), anterior insula (C), middle cingulate (D), primary somatosensory cortex (E), and the middle frontal gyrus extending into DLPFC (F). Statistical parametric maps, family-wise error corrected for multiple comparisons *P_FWE_* < 0.05, shown on average of 152 1 mm-resolution anatomical scans, normalized to MNI space. Corresponding in-plane MNI coordinate is shown below each image. Colorbar shows T-values at each voxel.

As expected, our fMRI GLM analysis of the Deviant > Standard contrast revealed extensive bilateral activations in primary somatosensory and parietal cortex. Within the right hemisphere, somatosensory activations covered 27.8% of area 2 and extended into areas of the intra-parietal cortex (IPC). The largest proportion of this activation was within area 2 (7.8% of cluster) followed by the IPC (6.1%). Consistent with previous oddball fMRI studies, we additionally observed significant bilateral activations in the dorsal mid-cingulate, anterior insula, and middle frontal gyrus extending into dorsolateral prefrontal cortex (BA 45). In the midbrain, we observed bilateral activations in dorsal-posterior somatosensory thalamus, and caudate nucleus. All anatomical labels and percent activations were determined using the SPM probabilistic anatomy toolbox. See Table 1 and Fig. 2 for a complete overview of these results.

### DCM results

**Fig. 3:**
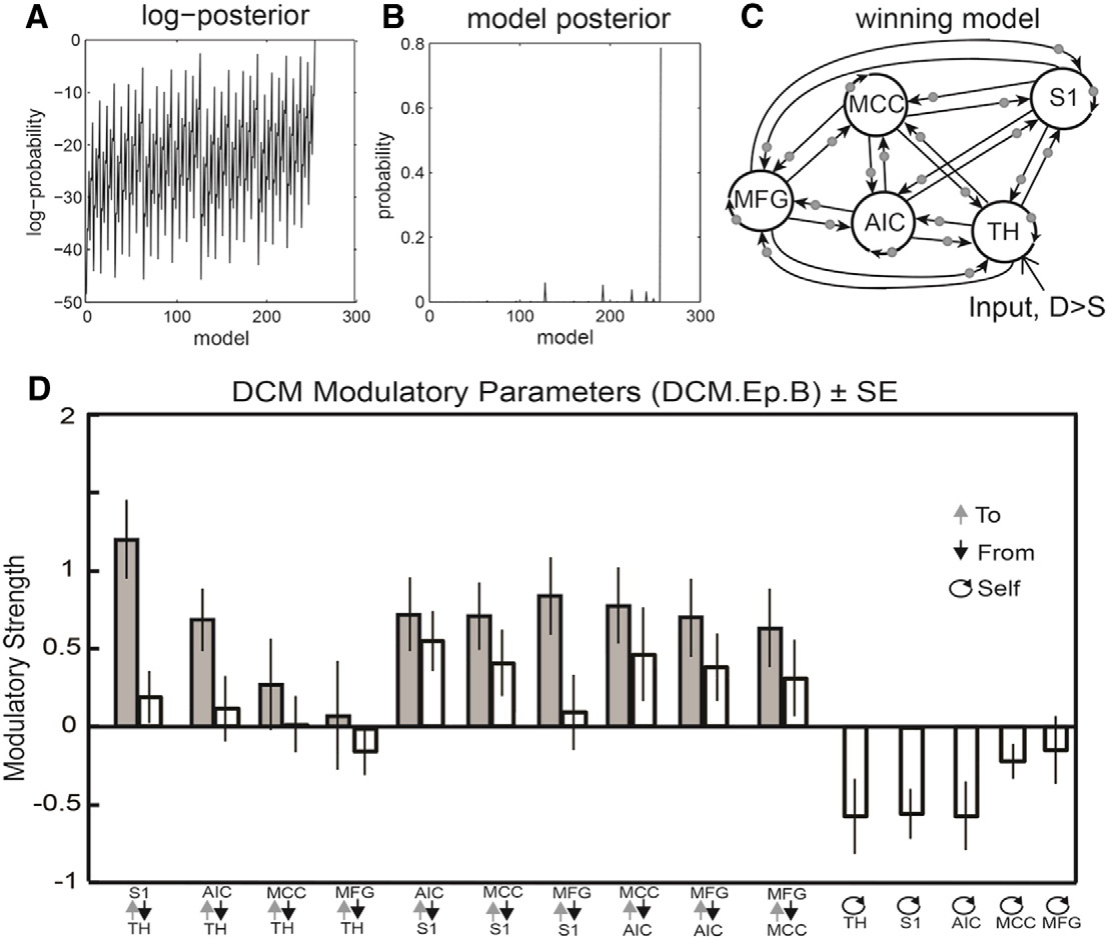
Post-hoc Bayesian model selection (panels A and B), winning model (panel C), and mean coupling parameter plots (panel D). (A) Top left panel depicts the range of log-posterior probability among all models examined. The top middle panel (B) shows the posterior probability for all tested models. Model 255 had the highest probability of 0.79. Model 128 was the next most probable with a posterior probability of 0.06, resulting in a Bayes factor of 13.17 for the full versus reduced model, corresponding to strong evidence that the full model was the best explanation for the measured data within the tested model space. (C) Depiction of the winning full model (Model 255, far right peak in Fig. 3B), grey circles indicate modulation by the Deviant > Standard contrast. (D) Bar plot depicting mean posterior parameter estimates for all modulatory (DCM.Ep.B) parameters across participants, indicating the strength in Hertz with which each connection was modulated by deviant > standard stimuli. Error bars depict standard error. Modulations of inhibitory self-connections are shown at the right hand side of the graph.

**Fig. 4:**
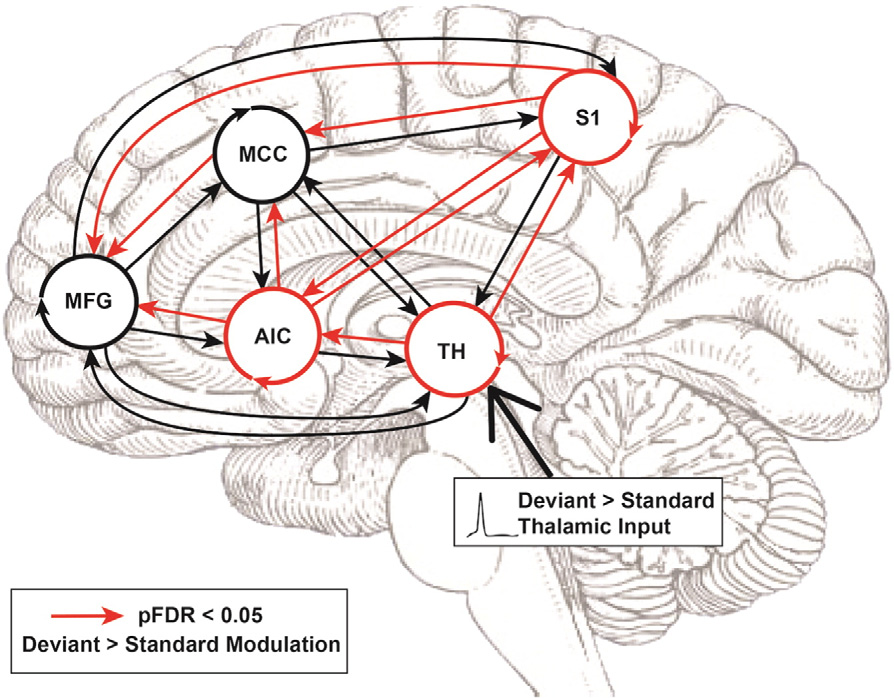
Full model and results of one-sample t-tests over estimated modulatory parameters. Red arrows depict results of one-sample t-tests over all 25 modulation parameters (inhibitory self-connections indicated by circular arrow around each region label). A general caudal to rostral flow increased effectivity connectivity in response to tactile deviants can be observed from thalamus (TH), and primary somatosensory cortex (S1), to anterior insula (AIC) and mid-cingulate (MCC), before reaching prefrontal cortex (middle frontal gyrus, MFG). In contrast to this feed-forward flow of modulatory influences, the AIC shows significant increases in both ‘forwards’ connections to cingulate and prefrontal cortex and ‘backwards’ connections with S1, indicative of error comparison. Interestingly, TH, AIC, and S1 self-connections are strongly disinhibited by tactile deviants. All p-values false discovery rate corrected for multiple comparisons, *P_FDR_* < 0.05.

Post-hoc model optimization found that the full model (M255, shown in Fig. 4B), with all extrinsic connections and modulations, had the highest posterior probability (pP = 0.79). The next most probable model was M128 with a posterior probability of 0.06; the Bayes factor discriminating these two models (pP_M255_/pP_M128_) was 13.17, corresponding to strong evidence for model 255 being the best explanation for the data (Penny et al., 2004). See Fig. 3 for an overview of the model selection results and plots illustrating connectivity strengths and their condition specific changes under the winning model.

One sample t-tests over all 25 modulatory parameters (at the between-subject level) revealed a general pattern of deviant-dependent increases in a forward (caudal to rostral) hierarchy, with significant increases in connectivity from TH to AIC and S1, from S1 to AIC, MCC, and MFG, from AIC to MCC and MFG, and from MCC to MFG (Fig. 4). In line with the hypothesis that AIC acts as a body-state comparator, the AIC exhibited both increased backward connectivity towards S1 and forward connectivity to the MCC and MFG. The AIC and S1 were the only regions to show reciprocal increases in connectivity. Additionally, significant modulations of the TH, AIC, and S1 self-connections were found, suggesting that somatosensory oddballs induce strong dis-inhibition of these regions.

**Fig. 5:**
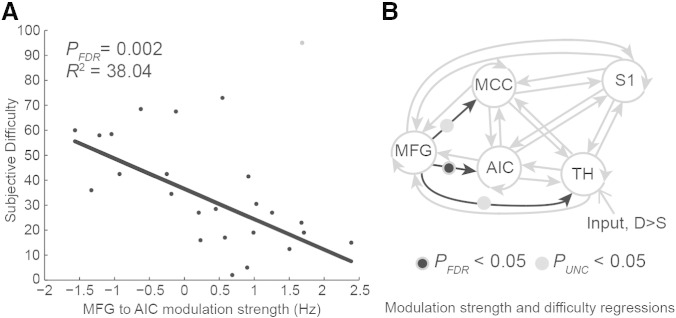
Robust regression analysis with self-reported difficulty for detecting stimulus changes predicting the strength of deviance-driven modulation of effective connectivity. Left panel A, participants with enhanced modulation of the backwards MFG to AIC connection by surprising touch stimuli reported easier discrimination of stimulus changes. Data points depict individual participants; points shaded grey indicate those receiving down weights > 2 SD from the mean weighting (leverage points). These results suggest that top-down prefrontal to AIC connectivity underlies awareness of unexpected tactile changes. Right panel B depicts results of robust regressions (Tukey's biweight) over 20 extrinsic connection modulation parameters each predicting subjective detection difficulty, P_FDR_ threshold < 0.05.

Our robust regression analyses found three condition-specific effects significantly predicted deviance-detection ratings (Fig. 5B); interestingly, all involved backwards connections (MFG to TH, MFG to MCC, and MFG to AIC). Only the MFG to AIC correlation survived FDR correction, with the Deviant > Standard modulation predicting 38% of the variance in subjective difficulty; *t*(1, 25) = − 3.47, *p*FDR = 0.002, R^2^ = 38.04 (Fig. 5A). Finally, in a control analysis, we calculated the average difference of self-reported intensity for high intensity–low intensity trials and repeated the above analysis using this measure of average stimulus differentiation. Only the MCC to AIC connection was significant at an uncorrected level (p = 0.0379), and did not survive multiple comparisons correction.

## Discussion

This study demonstrates that BOLD responses to surprising tactile stimuli are produced by increases in effective connectivity within a caudal-to-rostral ascending hierarchy of somatosensory, limbic, and prefrontal areas. In accordance with our hypothesis, anterior insula BOLD responses to deviants were explained by increases in the strength of both ascending influences on prefrontal and cingulate cortex and descending connectivity to the primary somatosensory area. Interestingly, individual differences in the modulation of descending connectivity predicted participant's subjective ratings of how easy it was to detect stimulus changes. Collectively these findings are consistent with the proposal that the anterior insula compares ascending body related sensations with top-down predictions to support tactile awareness. Here we consider the implications of these findings from the perspective of predictive coding and embodied active inference.

Previous fMRI studies of oddball responses in the visual, auditory, and tactile modalities report bilateral increases in BOLD activity in the thalamus (TH), primary sensory areas (e.g., S1/V1/A1), anterior insula (AIC), dorso-medial cingulate (MCC), and inferior and middle frontal gyrus (IFG, MFG), all of which are implicated in the present study (Downar et al., 2002, Garrido et al., 2009). Our mass-univariate results are thus highly consistent with a canonical oddball response in the tactile domain, confirming that unexpected touch is processed in the brain by a hierarchy of both modality-specific areas (posterior thalamus, S1) and a more cross-modal network of regions likely involved in orienting to salient events (AIC, MCC) and coordinating attention and cognitive control (IFG, MFG).

Deviance responses have been extensively studied using electrophysiological measures (e.g. M/EEG), which capture the well-characterized mismatch negativity (Garrido et al., 2009). Studies in the tactile domain have previously demonstrated mismatch negativity responses to sudden changes in stimulus location (Huang et al., 2005), intensity (Chen et al., 2008), and frequency (Kekoni et al., 1997). Crucially, a previous study using the RSOT modelled the tactile mismatch negativity as encoding Bayesian surprise, a computational measure of unsigned prediction error (Ostwald et al., 2012). Interestingly that study found that primary and secondary somatosensory cortices encoded an early (140 ms) stimulus-locked rise in Bayesian surprise whereas fronto-insular and cingulate sources showed a later response more associated with the representation of stimulus changes; i.e., salience.

Here, we observed strong activation of both S1 and AIC to tactile deviants, which was mediated by an increase in thalamic afference to both areas. These areas were in turn found to directly influence cingulate and prefrontal cortex. A plausible interpretation of both Ostwald's and our own results is that the AIC and MCC jointly monitor the precision (or reliability) of S1 responses. Precision has been linked to both perceptual salience and attention (Feldman and Friston, 2010), and – in predictive coding – enhances the influence of ascending prediction errors via the neuromodulatory regulation of post-synaptic cortical gain (Moran et al., 2013). Indeed, the AIC and MCC have been shown to encode expected precision or volatility (Iglesias et al., 2013, Schwartenbeck et al., 2014). More generally, the AIC and MCC are thought to form part of a ‘salience network’ that facilitates rapid orienting to important stimuli. Under predictive coding, salience (i.e. the selection of behaviourally relevant stimuli to attend to) can be operationalized as the precision-weighting of prediction errors by post-synaptic modulatory gain (Feldman and Friston, 2010, Friston et al., 2012). This formulation of hierarchical message passing is also consistent with our observation that deviancy decreased (inhibitory) intrinsic connectivity in lower regions of the tactile hierarchy. Physiologically, this corresponds to an increase in gain or excitability induced by unpredicted stimuli. This sort of effect is thought to underlie the mismatch negativity in the auditory domain, in which oddball stimuli increase precision at the sensory levels of the auditory hierarchy, relative to higher levels encoding prior expectations. This increased gain accounts for the larger evoked responses to tactile oddball stimuli observed here.

Consistent with the interpretation that the anterior insula monitors and regulates precision (i.e. reliability), we also found that deviancy signals bypassed S1 to directly modulate the AIC via thalamic afferents, in addition to an indirect route via S1. Interestingly, one previous study found that surprising painful stimuli bypass S1 to directly modulate the AIC (Liang et al., 2013). Thalamic cells are capable of firing in both tonic and ‘burst’ modes with the latter being important for the processing of salient events (Sherman, 2005, Sherman, 2007). Our finding that deviancy directly modulated the AIC, which in turn regulated down-stream S1 responses, suggests that the region may monitor the precision of thalamic outputs directly to enable fast awareness of and responding to critical events (e.g., pain, unexpected touch). This recurrent thalamic–insular–somatosensory loop is likely to be important for conscious awareness of tactile changes.

Indeed, recurrent neural activity in the somatosensory hierarchy has previously been shown to be important for somatosensory awareness (Auksztulewicz et al., 2012); in general such cortical-subcortical loops are thought to underlie conscious awareness (Dehaene et al., 2014). Here we found that strong recurrent connectivity between the AIC and somatosensory cortex supports the processing of tactile oddballs, and that individual differences in the strength of backwards influences from the PFC to AIC predicted the self-rated ease of detecting subtle stimulus changes. These findings together suggest that the AIC coordinates the global cortical processing of surprising bodily stimuli by linking lower-level sensory regions to more attention and salience-related areas in the prefrontal and cingulate cortex. In terms of predictive coding, this linking is likely to be mediated by modulating the precision of ascending prediction errors so that they exert greater influence on higher-level processing. As discussed above, an interesting possible interpretation is that the AIC supports the emergence of a deep hierarchical (c.f., global) workspace by monitoring and modulating the precision of ascending inputs (Bastos et al., 2012, Friston and Kiebel, 2009). Future studies will benefit from directly manipulating tactile precision, deviancy, and awareness in conjunction with computational modelling to address this possibility.

Finally, we observed a significant relationship between changes in backwards connectivity and difficulty ratings, wherein participants whose AIC was most strongly influenced by the PFC (during deviant processing) also reported easier detection of stimulus changes. This result links top-down effective connectivity to perceptual awareness, consistent with predictive coding simulations of attention in the visual domain (Feldman and Friston, 2010). This result thus establishes an intriguing link between the anterior insula and embodied active inference, providing criterion validity for our dynamic causal modelling results (Pennington, 2003). Interestingly, in participants reporting the lowest difficulty discriminating stimulus changes, the increases in PFC to AIC connectivity effectively completed a cortical–subcortical recurrent loop between somatosensory and prefrontal cortex (compare Fig. 4, Fig. 5), in line with a role for the AIC in coordinating a global neuronal workspace (Dehaene et al., 2014), and it may suggest an inherently embodied aspect to this workspace, mediated by connections through the AIC.

## Limitations and future directions

Although we interpret our results from the perspective of embodied active inference, interpretive caution is called for when relating effective connectivity results to computational schemes. Here, we used a biophysical model to capture directed neural influences between cortical and subcortical areas, which are not “computational” in the sense of directly modelling cognitive–functional mechanisms (Friston and Dolan, 2010). Our interpretation of these results is based on growing evidence that asymmetrical connections relate to specific computational variables (e.g. predictions, prediction errors and precision), and of repeated demonstrations that mismatch responses are better described by Bayesian prediction error minimization than other alternatives (Bastos et al., 2015, Garrido et al., 2007, Garrido et al., 2008, Lieder et al., 2013a). However, the possibility remains that another computational or functional theory may explain our results. For example, Lamme and others emphasize the necessity of feedback connections for attention and conscious perception (Bullier, 2001, Lamme et al., 1998). Here we also consider global workspace interpretations as complementary to our interpretation of recurrent S1-AIC activity underlying deviance perception. An important issue for future research will be to fit various computational models to the perception of bodily fluctuations, in order to evaluate how these models explain changes in effective connectivity and conscious perception (see for example Vossel et al., 2015).

Finally, although here we tentatively interpret subjective ratings of “how difficult was it to detect” stimulus changes as evidence for a role of top-down AIC modulation in perception, this finding must be treated with caution. Offline post-scan ratings can be subject to a variety of biases. An alternative interpretation of these results is that participants rated factors unrelated to level of sensory awareness, such as general cognitive effort. We find this particular interpretation unlikely however, because overall switch counts were extremely accurate (mean accuracy = 99%) and participants did not rate either condition as significantly more difficult to detect. In general participants rated an average detection difficulty of 35%, suggesting that it was generally easy to detect stimulus alternations. These observations make it unlikely that the subjective ratings are confounded by effort or vigilance, although previous studies do show that counting versus passive oddball tasks elicit differential prefrontal activity (Clark et al., 2001).

To further evaluate this finding, we explored whether a similar pattern of modulation would be observed for the overall difference in felt intensity rating for both stimulus conditions (see DCM Results). This analysis failed to find any effect of intensity ratings, suggesting that the top-down effect is specific to deviants themselves and not to the overall perception of (standard and deviant) stimulus intensity. To conclusively disambiguate this finding future research is needed; here our primary focus was on the pattern of insula connectivity evoked by oddballs, and our design was optimized accordingly. An effective future approach to address this question would be to modulate tactile expectations in a cued stimulus detection task coupled with subjective confidence ratings, potentially while also manipulating attention. Such an approach would better elucidate the role of attentional control and/or metacognitive report bias in modulating top-down connections and perceptual awareness of tactile changes (Fleming and Lau, 2014).

## Conclusion

This study demonstrates that attending to surprising tactile changes recruits a hierarchic ensemble of sensory, salience, and attention-related cortical areas. Our results illuminate the role of the insula in coordinating hierarchical processing of surprising tactile stimuli. If the AIC does coordinate dynamic interactions between these disparate neural processes, for example via monitoring and modulating precision, this would nuance the understanding of the “global neuronal workspace” theory of consciousness (Dehaene et al., 2014) by anchoring it in a fundamentally embodied network of sensory predictions. Embodied inference may thus provide a framework for understanding how particular predictive codes integrating bodily states and external sensory inputs give rise to self-awareness. Understanding how the insula coordinates this complex interaction, how various sensory channels are integrated and precision-weighted by contextual factors (Feldman and Friston, 2010), and how these processes relate to subjective experience and attention are critical areas for future research.

## Figures and Tables

**Table 1:** Significant Deviant > Standard BOLD activity.

Label	*k*	*P*_FWE_	*T*	MNI_XYZ_
R Anterior insula	403	< 0.001	11.16	36 20 1
R Caudate		< 0.001	8.74	12 8 4
R Inferior frontal gyrus (area 44)		< 0.001	8.15	54 8 19
R Primary somatosensory cortex (area 2)	344	< 0.001	10.06	48 − 37 46
R Intraparietal sulcus (hIP1–2)		< 0.001	9.65	39 − 43 43
R Intraparietal sulcus (hIP2)		< 0.001	9.18	39 − 52 52
L Intraparietal sulcus (hIP1–2)	493	< 0.001	9.91	− 39 − 49 43
L Primary somatosensory cortex (area 2)		< 0.001	9.58	− 45 − 43 49
L Intraparietal sulcus (hIP1–3)		< 0.001	9.13	− 33 − 55 49
L Pallidum	521	< 0.001	9.35	− 15 5 4
L Inferior frontal gyrus (area 44)		< 0.001	9.10	− 48 8 22
L Middle insula		< 0.001	8.08	− 42,11,− 2
L Anterior insula		< 0.001	8.27	− 30,23,− 2
L Temporal gyrus		< 0.001	8.49	− 48 8 − 2
R Middle frontal gyrus	160	< 0.001	8.45	36 50 22
R Middle frontal gyrus		< 0.001	7.76	42 44 25
R Middle frontal gyrus (DLPFC)		< 0.001	7.57	48 32 34
L Anterior mid-cingulate	141	< 0.001	8.45	− 3 20 40
R Anterior cingulate		0.006	6.62	9 17 25
L Supplementary motor area (area 6)		0.006	6.58	0 14 52
L thalamus (Th-prefrontal)	25	< 0.001	8.25	− 12 − 19 10
R Superior temporal gyrus	54	< 0.001	8.14	48 − 22 − 5
R Superior temporal gyrus		0.002	7.04	48 − 31 − 5
R Middle temporal gyrus		0.012	6.35	57 − 37 − 5
R Thalamus (Th-prefrontal)	51	< 0.001	8.10	12 − 16 10
R Thalamus (Th-prefrontal)		< 0.001	7.49	6 − 16 4
L Inferior parietal cortex (PF)	35	< 0.001	7.86	− 57 − 43 25
L Middle temporal gyrus		< 0.001	7.41	− 60 − 52 16
L Inferior frontal gyrus (area 44)	73	< 0.001	7.78	− 39 26 28
L Inferior frontal gyrus (area 45)		0.003	6.88	− 51 29 28
L Middle frontal gyrus		0.008	6.50	− 48 38 28
L Inferior parietal cortex (PFt, PFop)	33	< 0.001	7.59	− 57 − 22 34
R Middle frontal gyrus	39	< 0.001	7.36	27 11 58
R Middle frontal gyrus		0.010	6.42	39 5 55
L Middle frontal gyrus	58	< 0.001	7.32	− 36 41 22
L Middle frontal gyrus		0.002	6.94	− 36 50 16
L Prefrontal gyrus	17	0.002	7.11	− 45 2 52
R Superior temporal gyrus	32	0.002	7.05	48 − 40 13
R Inferior parietal cortex (PFcm)		0.012	6.33	57 − 40 25
R Inferior frontal gyrus (area 44)	12	0.005	6.67	51 14 40

## References

[bb0005] Andersson J.L.R., Hutton C., Ashburner J., Turner R., Friston K. (2001). Modeling geometric deformations in EPI time series. NeuroImage.

[bb0010] Apps M.A.J., Tsakiris M. (2014). The free-energy self: a predictive coding account of self-recognition. Multisensory integration, sensory substitution and visual rehabilitation. Neurosci. Biobehav. Rev..

[bb0015] Ashburner J., Friston K.J. (2005). Unified segmentation. NeuroImage.

[bb0020] Auksztulewicz R., Spitzer B., Blankenburg F. (2012). Recurrent neural processing and somatosensory awareness. J. Neurosci..

[bb0025] Baldi P., Itti L. (2010). Of bits and wows: a Bayesian theory of surprise with applications to attention. Neural Netw..

[bb0030] Barrett L.F., Simmons W.K. (2015). Interoceptive predictions in the brain. Nat. Rev. Neurosci..

[bb0035] Bastos A.M., Usrey W.M., Adams R.A., Mangun G.R., Fries P., Friston K.J. (2012). Canonical microcircuits for predictive coding. Neuron.

[bb0040] Bastos A.M., Vezoli J., Bosman C.A., Schoffelen J.-M., Oostenveld R., Dowdall J.R., De Weerd P., Kennedy H., Fries P. (2015). Visual areas exert feedforward and feedback influences through distinct frequency channels. Neuron.

[bb0045] Bullier J. (2001). Feedback connections and conscious vision. Trends Cogn. Sci..

[bb0050] Cerliani L., Thomas R.M., Jbabdi S., Siero J.C.W., Nanetti L., Crippa A., Gazzola V., D'Arceuil H., Keysers C. (2012). Probabilistic tractography recovers a rostrocaudal trajectory of connectivity variability in the human insular cortex. Hum. Brain Mapp..

[bb0055] Chang L.J., Yarkoni T., Khaw M.W., Sanfey A.G. (2013). Decoding the role of the insula in human cognition: functional parcellation and large-scale reverse inference. Cereb. Cortex.

[bb0060] Chen T.L., Babiloni C., Ferretti A., Perrucci M.G., Romani G.L., Rossini P.M., Tartaro A., Del Gratta C. (2008). Human secondary somatosensory cortex is involved in the processing of somatosensory rare stimuli: an fMRI study. NeuroImage.

[bb0065] Clark A. (2013). Whatever next? Predictive brains, situated agents, and the future of cognitive science. Behav. Brain Sci..

[bb0070] Clark V.P., Fannon S., Lai S., Benson R. (2001). Paradigm-dependent modulation of event-related fMRI activity evoked by the oddball task. Hum. Brain Mapp..

[bb0075] Craig A. (2003). Interoception: the sense of the physiological condition of the body. Curr. Opin. Neurobiol..

[bb0080] Craig A.D. (2005). Forebrain emotional asymmetry: a neuroanatomical basis?. Trends Cogn. Sci..

[bb0085] Dehaene S., Charles L., King J.-R., Marti S. (2014). Toward a computational theory of conscious processing. Curr. Opin. Neurobiol..

[bb0090] Dietz M.J., Friston K.J., Mattingley J.B., Roepstorff A., Garrido M.I. (2014). Effective connectivity reveals right-hemisphere dominance in audiospatial perception: implications for models of spatial neglect. J. Neurosci..

[bb0095] Downar J., Crawley A.P., Mikulis D.J., Davis K.D. (2002). A cortical network sensitive to stimulus salience in a neutral behavioral context across multiple sensory modalities. J. Neurophysiol..

[bb0100] Eickhoff S.B., Stephan K.E., Mohlberg H., Grefkes C., Fink G.R., Amunts K., Zilles K. (2005). A new SPM toolbox for combining probabilistic cytoarchitectonic maps and functional imaging data. NeuroImage.

[bb0105] Feldman H., Friston K. (2010). Attention, uncertainty and free-energy. Front. Hum. Neurosci..

[bb0110] Fleming S.M., Lau H.C. (2014). How to measure metacognition. Front. Hum. Neurosci..

[bb0115] Friston K. (2010). The free-energy principle: a unified brain theory?. Nat. Rev. Neurosci..

[bb0125] Friston K.J., Dolan R.J. (2010). Computational and dynamic models in neuroimaging. computational models of the brain. NeuroImage.

[bb0135] Friston K., Kiebel S. (2009). Predictive coding under the free-energy principle. Philos. Trans. R. Soc. Lond. B Biol. Sci..

[bb0140] Friston K., Penny W. (2011). Post hoc Bayesian model selection. NeuroImage.

[bb0130] Friston K.J., Williams S., Howard R., Frackowiak R.S.J., Turner R. (1996). Movement-related effects in fMRI time-series. Magn. Reson. Med..

[bb0120] Friston K., Adams R.A., Perrinet L., Breakspear M. (2012). Perceptions as hypotheses: saccades as experiments. Front. Psychol..

[bb0150] Garrido M.I., Kilner J.M., Kiebel S.J., Friston K.J. (2007). Evoked brain responses are generated by feedback loops. Proc. Natl. Acad. Sci..

[bb0145] Garrido M.I., Friston K.J., Kiebel S.J., Stephan K.E., Baldeweg T., Kilner J.M. (2008). The functional anatomy of the MMN: a DCM study of the roving paradigm. NeuroImage.

[bb0155] Garrido M.I., Kilner J.M., Stephan K.E., Friston K.J. (2009). The mismatch negativity: a review of underlying mechanisms. Clin. Neurophysiol..

[bb0160] Glover G.H., Li T.-Q., Ress D. (2000). Image-based method for retrospective correction of physiological motion effects in fMRI: RETROICOR. Magn. Reson. Med..

[bb0170] Huang M.-X., Lee R.R., Miller G.A., Thoma R.J., Hanlon F.M., Paulson K.M., Martin K., Harrington D.L., Weisend M.P., Edgar J.C., Canive J.M. (2005). A parietal–frontal network studied by somatosensory oddball MEG responses, and its cross-modal consistency. NeuroImage.

[bb0175] Hutton C., Bork A., Josephs O., Deichmann R., Ashburner J., Turner R. (2002). Image distortion correction in fMRI: a quantitative evaluation. NeuroImage.

[bb0180] Hutton C., Deichmann R., Turner R., Andersson J.L.R. (2004). Combined correction for geometric distortion and its interaction with head motion in fMRI. Proceedings of ISMRM.

[bb0185] Iglesias S., Mathys C., Brodersen K.H., Kasper L., Piccirelli M., den Ouden H.E.M., Stephan K.E. (2013). Hierarchical prediction errors in midbrain and basal forebrain during sensory learning. Neuron.

[bb0190] Kekoni J., Hämäläinen H., Saarinen M., Gröhn J., Reinikainen K., Lehtokoski A., Näätänen R. (1997). Rate effect and mismatch responses in the somatosensory system: ERP-recordings in humans. Biol. Psychol..

[bb0195] Lamme V.A., Supèr H., Spekreijse H. (1998). Feedforward, horizontal, and feedback processing in the visual cortex. Curr. Opin. Neurobiol..

[bb0200] Liang M., Mouraux A., Iannetti G.D. (2013). Bypassing primary sensory cortices—a direct thalamocortical pathway for transmitting salient sensory information. Cereb. Cortex.

[bb0205] Lieder F., Daunizeau J., Garrido M.I., Friston K.J., Stephan K.E. (2013). Modelling trial-by-trial changes in the mismatch negativity. PLoS Comput. Biol..

[bb0210] Lieder F., Stephan K.E., Daunizeau J., Garrido M.I., Friston K.J. (2013). A neurocomputational model of the mismatch negativity. PLoS Comput. Biol..

[bb0215] Lohmann G., Erfurth K., Müller K., Turner R. (2012). Critical comments on dynamic causal modelling. NeuroImage.

[bb0220] Lovero K.L., Simmons A.N., Aron J.L., Paulus M.P. (2009). Anterior insular cortex anticipates impending stimulus significance. NeuroImage.

[bb0225] Lund T.E., Madsen K.H., Sidaros K., Luo W.-L., Nichols T.E. (2006). Non-white noise in fMRI: Does modelling have an impact?. NeuroImage.

[bb0230] Menon V., Uddin L.Q. (2010). Saliency, switching, attention and control: a network model of insula function. Brain Struct. Funct..

[bb0235] Moran R.J., Campo P., Symmonds M., Stephan K.E., Dolan R.J., Friston K.J. (2013). Free energy, precision and learning: the role of cholinergic neuromodulation. J. Neurosci..

[bb0240] Ostwald D., Spitzer B., Guggenmos M., Schmidt T.T., Kiebel S.J., Blankenburg F. (2012). Evidence for neural encoding of Bayesian surprise in human somatosensation. NeuroImage.

[bb0245] Peirce J.W. (2007). PsychoPy—Psychophysics software in Python. J. Neurosci. Methods.

[bb0250] Pennington D.C. (2003). Essential Personality.

[bb0255] Penny W.D., Stephan K.E., Mechelli A., Friston K.J. (2004). Comparing dynamic causal models. NeuroImage.

[bb0260] Poldrack R.A. (2012). The future of fMRI in cognitive neuroscience. NeuroImage.

[bb0265] Rao R.P.N., Ballard D.H. (1999). Predictive coding in the visual cortex: a functional interpretation of some extra-classical receptive-field effects. Nat. Neurosci..

[bb0270] Rosa M.J., Friston K., Penny W. (2012). Post-hoc selection of dynamic causal models. J. Neurosci. Methods.

[bb0275] Schwartenbeck P., FitzGerald T.H.B., Mathys C., Dolan R., Friston K. (2014). The dopaminergic midbrain encodes the expected certainty about desired outcomes. Cereb. Cortex.

[bb0280] Seth A.K. (2013). Interoceptive inference, emotion, and the embodied self. Trends Cogn. Sci..

[bb0285] Seth A.K., Suzuki K., Critchley H.D. (2012). An interoceptive predictive coding model of conscious presence. Front. Psychol..

[bb0295] Sherman S.M. (2005). Thalamic relays and cortical functioning. Prog. Brain Res..

[bb0290] Sherman S.M. (2007). The thalamus is more than just a relay. Curr. Opin. Neurobiol. Sens. Syst..

[bb0300] Sridharan D., Levitin D.J., Menon V. (2008). A critical role for the right fronto-insular cortex in switching between central-executive and default-mode networks. Proc. Natl. Acad. Sci..

[bb0305] Ullsperger M., Harsay H.A., Wessel J.R., Ridderinkhof K.R. (2010). Conscious perception of errors and its relation to the anterior insula. Brain Struct. Funct..

[bb0165] van den Heuvel M.P., Kahn R.S., Goñi J., Sporns O. (2012). High-cost, high-capacity backbone for global brain communication. Proc. Natl. Acad. Sci..

[bb0310] Vossel S., Mathys C., Stephan K.E., Friston K.J. (2015). Cortical coupling reflects Bayesian belief updating in the deployment of spatial attention. J. Neurosci..

[bb0320] Worsley K.J., Marrett S., Neelin P., Vandal A.C., Friston K.J., Evans A.C. (1996). A unified statistical approach for determining significant signals in images of cerebral activation. Hum. Brain Mapp..

[bb0315] Worsley K.J., Liao C.H., Aston J., Petre V., Duncan G.H., Morales F., Evans A.C. (2002). A general statistical analysis for fMRI data. NeuroImage.

